# A Bayesian perspective on severity: risky predictions and specific hypotheses

**DOI:** 10.3758/s13423-022-02069-1

**Published:** 2022-08-15

**Authors:** Noah van Dongen, Jan Sprenger, Eric-Jan Wagenmakers

**Affiliations:** 1 University of Amsterdam, Amsterdam, Netherlands; 2 University of Turin, Turin, Italy

**Keywords:** Statistical test, Bayes factors, Null hypothesis significance testing, Severity, Error statistics, Karl Popper, Deborah Mayo

## Abstract

A tradition that goes back to Sir Karl R. Popper assesses the value of a statistical test primarily by its *severity*: was there an honest and stringent attempt to prove the tested hypothesis wrong? For “error statisticians” such as Mayo (1996, 2018), and frequentists more generally, severity is a key virtue in hypothesis tests. Conversely, failure to incorporate severity into statistical inference, as allegedly happens in Bayesian inference, counts as a major methodological shortcoming. Our paper pursues a double goal: First, we argue that the error-statistical explication of severity has substantive drawbacks; specifically, the neglect of research context and the specificity of the predictions of the hypothesis. Second, we argue that severity matters for Bayesian inference via the value of specific, risky predictions: severity boosts the expected evidential value of a Bayesian hypothesis test. We illustrate severity-based reasoning in Bayesian statistics by means of a practical example and discuss its advantages and potential drawbacks.

*What, then, is the end of an explanatory hypothesis? Its end is, through subjection to the test of experiment, to lead to the avoidance of all surprise and to the establishment of a habit of positive expectation that shall not be disappointed.* C.S. Peirce (1931)[CP 5.197]

The Bayesian framework for statistical inference—expressing one’s uncertainty about hypotheses and parameters by means of subjective probabilities and updating them through Bayes’ theorem—is increasingly popular in psychology (e.g., Rouder et al., 2009; Lee & Wagenmakers, 2013; Vandekerckhove et al., 2018). This popularity is easy to explain: Bayesian inference is based on a general theory of uncertain reasoning, its basic principles are simple and easily remembered, and statistical evidence is quantified by means of relative predictive performance (i.e., Bayes factors); moreover, Bayesian inference avoids the problems with the interpretation of *p*-values, and statistical inferences are connected to our beliefs and the practical consequences of our decisions (e.g, Jeffreys, 1961; Jeffrey,^1971^, Savage, 1972; Bernardo & Smith, 1994; Lindley, 2000; Howson & Urbach, 2006; Evans, 2015; Morey et al., 2016; Sprenger & Hartmann, 2019).

A major objection to Bayesian inference consists in its apparent neglect of the role of severe hypothesis tests in scientific inference. For champions of severe testing like Sir Karl R. Popper (1963, 1959/2002), hypotheses are not confirmed by their predictive performance or their agreement with available data. Rather, they count as confirmed only if they have survived repeated and stringent attempts to prove them wrong. What matters for the status of a hypothesis is its *probative value*, or, in other words, whether it has “proved its mettle” (Popper, 1959/2002, p. 264). Via the work of methodologists and statisticians such as Ronald A. Fisher (1935, 1956) and Paul Meeh (1978, 1986, 1990a, 1990b), Popper’s idea has also exerted a profound influence on statistical practice in social science.

Recently, Deborah Mayo (1996, 2018) has developed a philosophy of statistical inference called *error statistics*, based on the concept of severe testing. Mayo states explicitly that data should only count as evidence for a claim C if they resulted from a real and severe test; otherwise we have “BENT: Bad Evidence—No Test” (p. 5 ; Mayo, 2018). Mayo’s account deals primarily with controlling (Type I and II) error rates in inference and is therefore frequentist in nature (cf. Neyman & Pearson, 1933; 1967; Neyman, 1977; Mayo, 1996). One of the key objectives of this paper is to provide a critical analysis of Mayo’s account and to assess the prospects of error statistics for application in social science.

The other objective consists in outlining how Bayesians can implement the ideas of severe testing and error control into their inference framework. Prima facie, neither the posterior probability of a hypothesis nor the Bayes factor seems to depend on the severity of a test, or the extent to which one has tried to prove the theory wrong. Specifically, our paper addresses the following questions:

1. Should Bayesians care about severity?
2. How can Bayesians account for the value of severity in inference?
3. Is severity more naturally accounted for in frequentist than in Bayesian inference?

We will defend an affirmative answer to the first question, sketch a constructive answer to the second question and give a negative answer to the third question. However, note that none of these positions is self-evident.

With respect to Q1, many Bayesians deny that severity should matter at all in inference. They refer to the *Likelihood Principle*: all the evidence that an experiment provides about an unknown quantity is expressed by the likelihood function of the various hypotheses on the observed data (Birnbaum, 1962; Edwards et al., 1963; Berger & Wolpert, 1984). “Consequently the whole of the information contained in the observations that is relevant to the posterior probabilities of different hypotheses is summed up in the values that they give to the likelihood” (Jeffreys, 1961, p. 57). Therefore, the severity of a test, as expressed by whether the hypothesis of interest stood a risk of being refuted (e.g., whether unobserved data could have proven it wrong), cannot enter the statistical evaluation of an experiment. According to this line of response, Popper, Mayo and other defenders of severe testing are just mistaken when they believe that severity should enter the (post-experimental) assessment of a theory. Much of the “statistics wars” Mayo (2018, p. xi) between Bayesians and frequentists have revolved around this controversy (see also ; Mayo & Kruse, 2001; Mayo, 2010). Our paper, by contrast, acknowledges Popper’s and Mayo’s argument that severity needs to be accounted for by an adequate logic of scientific, and statistical, inference.

With respect to Q2, Bayesians have only recently started to care about severity and to explain its role in Bayesian inference (Vanpaemel, 2010; Lee & Vanpaemel, 2018; Vanpaemel, 2019; 2020; Dienes, 2021). In particular, Vanpaemel (2020) argues that severity does not (only) consist in making precise predictions: a severe test has to rule out *plausible* outcomes, that is, outcomes with a high prior predictive probability (we engage with Vanpaemel’s positions in a later section). Our own account highlights how the specificity of a hypothesis and its predictions boost severity by raising the expected evidential value of the experiment, and how error control can be embedded naturally into Bayesian inference. Specifically, we construe severity within the Bayesian framework as the *specificity of the prediction of a hypothesis in relation to the potential data that could be observed*. The value of severity is reflected in the *expected evidential value* of a test, quantified by the expected absolute log-Bayes factor (Lindley, 1956; Good, 1979; Cavagnaro et al., 2010; Schönbrodt and Wagenmakers, 2018; Stefan et al., 2019). In addition, we show, in line with Vanpaemel, how prior probability distributions connect scientific theory with a statistical model, and how priors contribute to the severity of a test (Vanpaemel, 2010; 2019).

Finally, with regard to Q3, we argue that the error-statistical explication of severity faces considerable conceptual challenges. Since error statistics enjoys increasing popularity in the psychological science community (see, e.g., Haig 2020or the https://richarddmorey.shinyapps.io/severity/), we believe that this negative argument is an important contribution to the debate. In other words, we show that a statistical practitioner who cares about severe testing need not be a frequentist. Severity is a concept that can be integrated equally well, or even better, into the framework of Bayesian inference.

## Theoretical background: scientific theories and severe tests

Scientific theories are imperfect descriptions and explanations of reality. They allow us, with a certain degree of accuracy, to predict future observations or to uncover structure in available data. According to Popper (1959/2002), the value or *empirical content* (p. 96) of a scientific theory lies in the combination of its *universality* and its *precision* (i.e., generality to what it pertains and specificity of what it predicts/explains; pp. 105–106). A theory has high empirical content when it has a vast scope in which many observations are possible in principle (e.g., all planetary motions in the universe); yet only a few of these possible observations are consistent with the theory (e.g., ellipses around a center of gravity). This is consistent with the common intuition that a successful risky prediction is more impressive than a successful vague prediction.

Meehl (1978) expressed this intuition with an example of two meteorological theories that both make predictions on next month’s weather. Theory A predicts: in Turin it will rain on three days in the next month. Theory B predicts: in Turin it will rain on the third, fourth, and the seventh day of next month. We expect that most people agree with Meehl that a success of Theory B’s prediction is more impressive than the success of Theory A’s prediction. This difference can be made explicit in terms of its *falsifiablity* (Popper, 1959/2002, p. 96): Theory B’s prediction is only correct in ${30 \choose 1}=1$ out of 2^30^ possibilities, while Theory A’s prediction is correct in ${30 \choose 3}=4060$ out of 2^30^ possibilities. What is important for the standing of a scientific theory is not its ability to fit the data, but how specific it is with respect to all possible data (see also ; Roberts and Pashler, 2000). Popper (1959/2002) gave the examples of Freudian psychoanalysis and Marxist sociology as theories that could fit any pattern of data, thus being low in empirical content and even unscientific. For these ‘theories’, both the presence and absence of a personality trait or both presence and absence of worker unrest could be considered as consistent with the theory.^1^

Competing theories are falsifiable to the extent that they make sharply contrasting predictions. The outcome of a test should either favor one theory and contradict the other, or vice-versa (i.e., strong inference: Platt 1964 or *experimentum crucis* as defined by Hooke: Lohne 1968):

The scientific method is always comparative and there are no absolutes in the world of science. It follows from this comparative attitude that a good theory is one that enables you to think of an experiment that will lead to data that are highly probable on [the theory], highly improbable on [the complement of the theory], or vice versa, so that the likelihood ratio is extreme and your odds substantially changed. (p. 209 ; Lindley, 2006)

In modern mathematical language, this means that one needs to maximize the expected information gain regarding the assessment of the competing theories (Oaksford and Chater, 1994; Myung & Pitt, 2009; Myung et al., 2013). A prime example is Eddington’s 1919 test of Einstein’s General Theory of Relativity (GTR) versus Newton’s classical theory of gravity (Dyson et al., 1920). Both theories make sharp mutually exclusive predictions about the degree to which passing massive bodies, like our sun, bend light from distant sources, like other stars. The predictions of GTR were famously verified by Eddington during the 1919 solar eclipse.

The benefits to science from increasing the falsifiability of theories and riskiness of predictions have been presented in numerous critiques of social science methodology (e.g., Meehl, 1978; 1986; 1990a, b; Roberts & Pashler, 2000). In the case of GTR vs. Newtonian mechanics, the specificity of the predictions of a theory is tightly linked to the *capacity for testing it severely* (and thus, to its falsifiability). The higher the proportion of possible outcomes that are consistent with a theory, the less informative the theory becomes. For example, it is hard to falsify the rather uninformative theory that it will rain in Amsterdam on some days in the next year. On the opposite side, we have highly informative theories, with a limited set of parameters whose values are fixed and interrelated. An excellent example of such a theory is GTR. This theory makes specific predictions about time dilatation in GPS satellites relative to the earth’s surface, improving navigational accuracy. The more informative a theory is, the more falsifiable it is, because fewer possible observations are consistent with it. Only such informative theories can be severely tested:

We may say that to make predictions with great accuracy increases the probability that they will be found wrong, but in compensation they tell us much more if they are found right. [...] The best procedure, accordingly, is to state our laws as precisely as we can, while keeping a watch for any circumstances that may make it possible to test them more strictly than has been done hitherto. (pp. 39–40; Jeffreys, 1973)

We now investigate how severity manifests itself in various statistical frameworks, starting with Mayo’s error statistics.

## Severity in error statistics

At first glance there is a striking resemblance between Popper’s emphasis on the severe testing of scientific hypotheses and *null hypothesis significance tests* (NHST). Typically, at the center of NHST there is a *point null hypothesis* postulating that a parameter takes a precise value (e.g., the mean of a population, *H*_0_ : *μ* = *μ*_0_)—corresponding to absence of a causal effect in an experimental intervention, equality of two medical treatments, and so on. When the measured divergence from the null hypothesis exceeds a given threshold (e.g., the observed test statistic falls in the most extreme 5% of the probability density function), the null hypothesis is *rejected* and the statistical analysis reports *statistically significant* results against the null. Otherwise, no conclusion is drawn.

At first glance, the NHST methodology squares well with Popper’s falsificationism. First, the point null hypothesis is maximally falsifiable, because it commits to a single point value for the effect. Second, the idea of submitting the point null to a severe test and rejecting it when it fails to explain the data, has a distinct Popperian note. However, the scientific theory that we would like to submit to a severe test is usually *not* the null hypothesis (see also ; Rouder et al., 2009; Gallistel, 2009; Sprenger & Hartmann, 2019, ch. 9). Rather, we would like to severely test the hypothesis that there *is* a meaningful effect: that a medical drug is better than a placebo, that playing a musical instrument makes people happier, that video games improve reasoning skills, and so on. This “alternative” hypothesis usually takes the generic form *H*_1_ : *μ* > *μ*_0_ (or even *H*_1_ : *μ*≠*μ*_0_). It does not commit the scientist to precise predictions; rather it states that the unknown parameter lies in a *range* of values. NHST does not explain how such hypotheses can be tested severely; nor does it give a general definition of severity in statistical inference.

Mayo’s (1996, 2018) *error-statistical approach* addresses this problem and explicates how parametric hypotheses can be severely tested in general. Error statistics follows Popper’s perspective on theory testing as the ability to detect and control error between data and hypothesis. The standing of a theory is primarily determined by the severity of the tests it has survived. The extent to which the theory has proved its mettle constitutes statistical evidence. Mayo (2018, p. 14) states this explicitly in her Strong Severity Principle:

**Severity Principle (strong):** We have evidence for a claim C just to the extent it survives a stringent scrutiny. If C passes a test that was highly capable of findings flaws or discrepancies from C, and yet none or few are found, the passing result, x, is evidence for C.

*Prima facie*, two important elements of severe testing are missing in the Severity Principle (in this formulation). First, Mayo’s perspective does not make explicit essential elements of severe testing that Popper stressed: the universality of the tested hypothesis *H* and the specificity of the predictions it makes (Popper, 1959/2002, p. 266). Nowhere in Mayo’s severity principle or in the specifications discussed below is the riskiness or specificity of a hypothesis, and the predictions it makes, incorporated as a requirement for a severe test (Meehl, 1978; 1986; 1990b, a; Roberts & Pashler, 2000).^2^

Second, without explicit reference to alternatives to the claim, it is not clear how a claim can survive “stringent scrutiny” or when a test is “highly capable” of finding differences. For example, a stringent scrutiny of the claim C: “90% of all swans are white” requires only a single swan if the alternative claim is “all swans are black”. When the alternative is that “fewer than 80% of all swans are white”, however, the observation of a single white swan is relatively uninformative, and a large sample of swans is needed to discriminate between both hypotheses. If C encompasses all that is possible (except ¬C), no test —no matter how probative it usually is— will be capable of severely testing C. This suggests that the Severity Principle should not be interpreted in absolute, but in *relative terms*, comparing the claim to the most plausible and most severely tested competing hypotheses (e.g., as done by Eddington in the 1919 solar eclipse experiment). This contrastive aspect of severe testing is missing in Mayo’s formalization.

This problem comes into sharper focus when we consider Mayo’s operationalization of the Severity Principle Mayo (2018, p. 92):

**Severity Requirement:** for data to warrant hypothesis *H* requires not just that (S-1) *H* agrees with the data (*H* passes the test), but also (S-2) with high probability, *H* would not have passed the test so well, were *H* false.

Suppose that the hypothesis of interest is *μ* ≥ *μ*_0_ + *δ*, where *δ* > 0 expresses the effect size relative to the default value *μ*_0_. Mayo calls the hypothesis of interest the ‘Claim’ and indicates it with *C*. The application of (S-1) is then similar to the Neyman-Pearson (and NHST) approach (see Mayo 2018, p. 142): *C* passes a statistical test when *H*_0_ : *μ* ≤ *μ*_0_ is rejected at the pre-specified Type I error level *α*.^3^ This happens when the probability of the observed data, or data deviating more extremely from *H*_0_, is lower than *α* (i.e., *p*(*d*(*X*) ≥ *d*(*x*);*H*_0_) < *α*), where the statistic *d* measures divergence of the data from *H*_0_).

To assess the evidence in favor of *C*, Mayo applies (S-2) and computes a severity function (*SEV*: see the corresponding https://richarddmorey.shinyapps.io/severity/). The severity function takes as arguments the statistical test, the observed data, and the target hypothesis *C*. The severity function outputs the probability of obtaining the observed data *x* or data closer to *H*_0_ and deviating more from *C*, if *C* were false. As a representative of the negation of *C*, we choose the point hypothesis in *C* that is closest to *H*_0_, which is *μ* = *μ*_0_ + *δ*. If this hypothesis is rejected with test *T* based on data *x*, the severity with which *C* has passed the test is defined as

$$ \begin{array}{@{}rcl@{}} SEV(T, x, C) &=& p(d(X) \leq d(x); \neg C)\\ &=& p(d(X) \leq d(x); \mu = \mu_{0} + \delta), \end{array} $$

which is (a lower bound on) the probability of observing the actually observed data, or data more deviating from *C*, in the direction of *H*_0_ if *C* were false.^4^

To show how the severity function works in practice, we borrow a simple example from (Mayo, 2018, pp. 142–144). The example concerns the scenario of an “accident at a water plant” (p. 142) where a leak in the cooling system is discharging water into the ecosystem. The cooling system is “meant to ensure that the mean temperature of discharged water stays below the temperature that threatens the ecosystem, perhaps not much beyond 150 degrees Fahrenheit.”

The question is, whether the temperature of the water high enough to constitute an ecological disaster that requires counter measures beyond mere repair of the cooling system. When the cooling system is working properly, the temperature of the water discharged into the ecosystem is around 150 degrees Fahrenheit and a temperature of 153 or higher is considered a “full-on emergency for the ecosystem” (p. 143). A series of 100 water measurements are taken at random time periods and their sample mean $\overline {x}$ is computed. The standard deviation is known and when the cooling system is working properly, the distribution of means from samples of 100 measurements is captured by $\overline {X} \sim N(\mu =150, 1)$. Mayo supposes that initially the test concerns “*H*_0_ : *μ* ≤ 150vs.*C* : *μ* > 150” (p. 142). She sets the Type I error rate to 2.5% (*α* = 0.025), thus rejects “*H*_0_ (infer there’s an indication that *μ* > 150) iff $\overline {X}\geq 152$” (p. 142).^5^

**Fig. 1:**
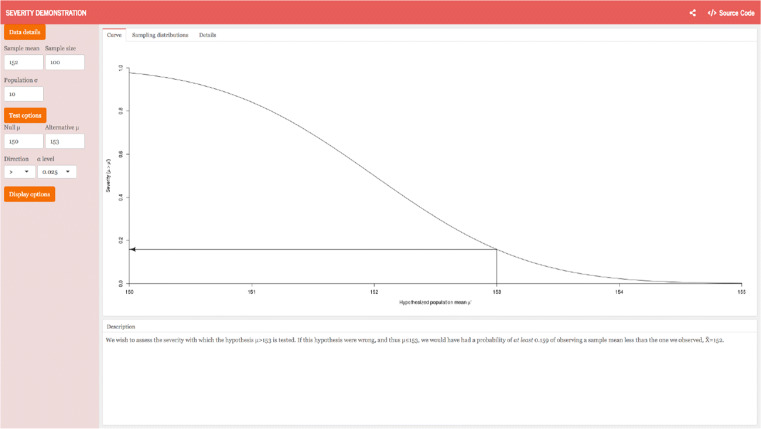
*SEV* results for the original water plant example. In this case, *H*_0_ : *μ* = 150, *C* : *μ* = 153, $\overline {x}=152$, and *SEV* = 0.159. This image is a screenshot from the *Severity Demonstration application*. This Shiny App was developed by Morey (2020) and can be accessed via https://richarddmorey.shinyapps.io/severity/?mu0=150&mu1=153&sigma=10&n=100&xbar=152&xmin=150&xmax=155&alpha=0.025&dir=%3E

In this scenario, we observe a sample mean of 152 degrees Fahrenheit and thus reject *H*_0_, fulfilling (S-1) of the Severity Requirement. According to the severity rationale, we are warranted in concluding that *μ* > 150, because “[w]ere the mean temperature no higher than 150, then over 97% of the time their method would have resulted in a lower mean temperature than observed” (p. 143). However, we are primarily concerned with the extent to which these data indicate that the water has reached devastating temperatures of 153 Fahrenheit or higher (*μ* ≥ 150 + *δ*;*δ* = 3). According to the Severity Requirement (S-2), we need the probability of observing our mean temperature of 152 or lower if the actual temperature is equal the critical threshold of 153 degrees. This can be calculated with the *SEV* function, which is $p(\overline {X} \leq 152; \mu = 153) \approx 0.16$. From these results, one can conclude that one does not have evidence for the claim that the water has reached temperatures of 153 degrees Fahrenheit or higher. Namely, the “severity principle blocks *μ* > 153 because it is fairly probable (84% of the time) that the test would yield an even larger mean temperature than we got, if the water samples came from a body of water whose mean temperature is 153” (p. 144). Thus, the results do not meet requirement (S-2) and the hypothesis that the temperature of the water is 153 degrees or higher does not pass a severe test (see Fig. 1).

**Fig. 2:**
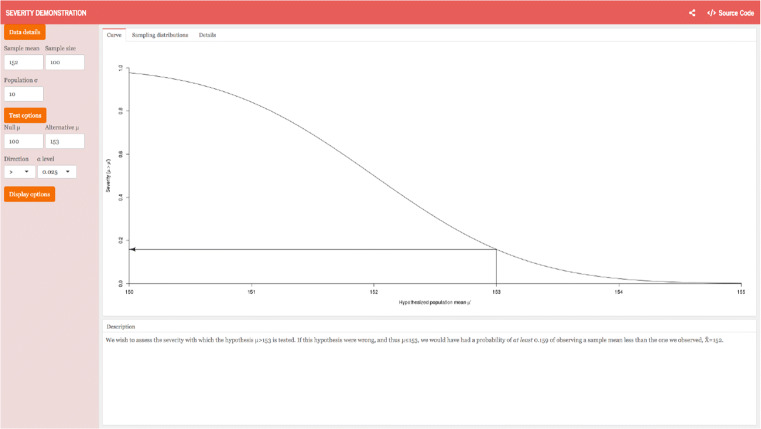
*SEV* results for the original water plant example. In this case, *H*_0_ : *μ* = 100, *C* : *μ* = 153, $\overline {x}=152$, and *SEV* = 0.159. This image is a screenshot from the *Severity Demonstration application*. This Shiny App was developed by Morey (2020) and can be accessed via https://richarddmorey.shinyapps.io/severity/?mu0=100&mu1=153&sigma=10&n=100&xbar=152&xmin=150&xmax=155&alpha=0.025&dir=%3E

With this depiction, a consequence of the second problem described above comes into view; the severity principle operationalized as the *SEV* function implies a curious, perhaps outright problematic concept of statistical evidence. In the water plant example, the observed mean temperature of 152 degrees Fahrenheit is not considered evidence that the water has reached the dangerous temperature of 153 degrees ($SEV(T, \overline {x},\mu > 153) \approx 0.16$). However, this result is *independent of what is considered the normal or default state of affairs*. In Mayo’s example, this is 150 degrees Fahrenheit, but this value is irrelevant for her *SEV* function as long as the observed mean is sufficiently high to reject the null hypothesis. Suppose that we had observed the same mean temperature of 152 degrees Fahrenheit, though the normal mean temperature is 100 degrees. Then one would again reject *H*_0_ and meet requirement (S-1); one would again not meet (S-2) and draw the same conclusion as before (see Fig. 2).

However, when normal temperatures are around 100 degrees, you observe a mean temperature of 152 with a standard error of 1, and an “full-on emergency for the ecosystem” is imminent when the temperature is 153, it is clear that counter measures are acutely required. Evidence must be evaluated in context and relative to the competing or default hypotheses. The error-statistical analysis of the case goes against any intuition one might have about the concept of severity, treatments of this concept by other philosophers and methodologists (e.g., Popper, 1959/2002; Meehl, 1990b; 2005; Roberts and Pashler, 2000), and even against a reasonable interpretation of Mayo’s Severity Principle (Mayo, 2018, p. 14)

For this reason, we disagree that Mayo’s Severity Principle and Severity Requirement (as explicated by the Severity Function) are good operationalizations of the function of severity in scientific inference and statistical testing. To recapitulate, our main criticism are (1) any theory of severe testing must be sensitive to what is considered the normal or default state of affairs, and Mayo’s theory does not; (2) more generally, Mayo’s theory neglects that the severity of a test is a function of the degree of similarity between the competing hypotheses, and the specificity of their predictions. Mayo’s explications of severity in statistical inference are decoupled from the scientific context in which the data are collected.

## Severity in Bayesian inference

In Bayesian inference, an observation *x* supports hypothesis *H* if and only if *x* raises the subjective probability of *H* (e.g., Carnap, 1950; Horwich, 1982; Howson & Urbach, 2006; Evans, 2015; Sprenger & Hartmann, 2019):

$$ \begin{array}{@{}rcl@{}} p({H} \mid x) > p({H}). \end{array} $$

Equivalently, in the case of a null hypothesis *H*_0_ and an alternative *H*_1_, *x* is evidence for *H*_1_ if and only if *x* is better predicted under *H*_1_ than under *H*_0_, i.e., if the *Bayes factor* BF_10_ exceeds 1:

$$ \text{BF}_{10} (x) := \frac{p(x \mid {H}_{1})}{p(x \mid {H}_{0})} > 1. $$

The higher the Bayes factor, the stronger the evidence for *H*_1_, and the closer it is to zero, the stronger the evidence for *H*_0_. See Table 1 for a conventional classification of Bayes factors.

**Table 1:** Classification of Bayes factors according to Lee and Wagenmakers (2013), adjusted from Jeffreys (1961)

Bayes Factor *BF*_10_	Interpretation
> 100	Extreme evidence for *H*_1_
30–100	Very strong evidence for *H*_1_
10–30	Strong evidence for *H*_1_
3–10	Moderate evidence for *H*_1_
1–3	Anecdotal evidence for *H*_1_
1	No evidence for either hypothesis
1/3–1	Anecdotal evidence for *H*_0_
1/3–1/10	Moderate evidence for *H*_0_
1/10–1/30	Strong evidence for *H*_0_
1/30–1/100	Very strong evidence for *H*_0_
< 1/100	Extreme evidence for *H*_0_

The posterior probability of a theory *H*_0_ given data *x* is calculated as *p*(*H*_0_|*x*) = *p*(*H*_0_) ⋅ *p*(*x*|*H*_0_)/*p*(*x*). This allows us to write the posterior odds in favor of *H*_0_ over *H*_1_ as

$$ \frac{p({H}_{0}\mid x)}{p({H}_{1} \mid x)} = \frac{p({H}_{0})}{p({H}_{1})}\cdot \frac{p(x \mid {H}_{0})}{p(x \mid {H}_{1})}. $$

Thus, keeping the prior probability *p*(*H*_0_) fixed, the posterior probability of *H*_0_ will be the larger (1) the better *H*_0_ predicts *x* and (2) the more surprising (worse predicted) *x* is under *H*_1_ (e.g., Roberts and Pashler, 2000; Howson & Urbach, 2006).

By construction, the Bayes factor is relative to an explicit choice of context, i.e., the alternative hypothesis. Quite often, this alternative hypothesis will depend on the prior distribution of the parameter of interest, e.g., when we test a point null hypothesis *H*_0_ : *μ* = *μ*_0_ against an unspecific alternative *H*_0_ : *μ*≠*μ*_0_. However, as argued by Vanpaemel (2010), this property of Bayes factors is a virtue rather than a vice: the prior distribution expresses, after all, our theoretical expectations and predictions. A scientist using Bayesian inference needs to think about the prior distribution in advance, strengthening the link between scientific theorizing and statistical analysis whose absence has often been named as a cause of the lack of reliability and replicability of psychological research (compare ; Meehl, 1967; Ioannidis, 2005; Dienes, 2021).

parAt first glance, severity-related aspects appear to be lacking in the Bayesian paradigm: the Bayes factor only depends on the probability of the data in light of the two competing hypotheses. As Mayo emphasizes (e.g., Mayo and Kruse, 2001; Mayo, 2018), the Bayes factor is insensitive to variations the sampling protocol that affect the error rates, i.e., optional stopping of the experiment. The Bayes factor only depends on the actually observed data, and not on whether they have been collected from an experiment with fixed or variable sample size, and so on. In other words, the Bayesian ex-post evaluation of the evidence stays the same regardless of whether the test has been conducted in a severe or less severe fashion.

We agree with this observation, but we believe that the proper place for severity in statistical inference is in the choice of the tested hypotheses (are they specific? are they sufficiently contrastive?), and in the experimental design. Bayesians cash out severity by ensuring that an experiment gives positive answers to the two questions below:

Evidential Value: Is it probable to *obtain strong, discriminatory evidence* from this particular experiment?
Error Control: Does the design of the experiment *limit the probability of finding misleading evidence*? That is, does it limit the probability of making an erroneous inference?

These properties also depend on the experimental design as a whole (e.g., the chosen sample size), but specifically on the choice of the tested hypotheses:

Specificity: Do the competing hypotheses *H*_0_ and *H*_1_ make *specific predictions*? That is, do they rule out, or make implausible, a large proportion of possible outcomes?
Contrastivity: Are the predictions of hypothesis *H*_0_ and *H*_1_ *sufficiently different* that the experiment can discriminate between them?

In other words, the specificity and contrastivity of a hypothesis contributes to the severity of a test by increasing its evidential value and controlling error in inference. We will now discuss these concepts in detail.

### Specificity and evidential value in Bayesian inference

For a test to be severe, the tested hypothesis needs to *impose substantial restrictions on the range of potential data* that are consistent with it. This is a basic ingredient of severe testing that has been retained both by philosophers such as Popper and psychological science methodologists. For example, Roberts and Pashler (2000, p. 359) argue that a good fit between data and model does not have to be convincing as evidence when the parameter values of the model are fully adjustable to accommodate the data:

Theorists who use good fits as evidence seem to reason as follows: if our theory is correct, it will be able to fit the data; our theory fits the data; therefore it is more likely that our theory is correct. *However, if a theory did not constrain possible outcomes, the fit is meaningless.* (Italics added for emphasis)

**Fig. 3:**
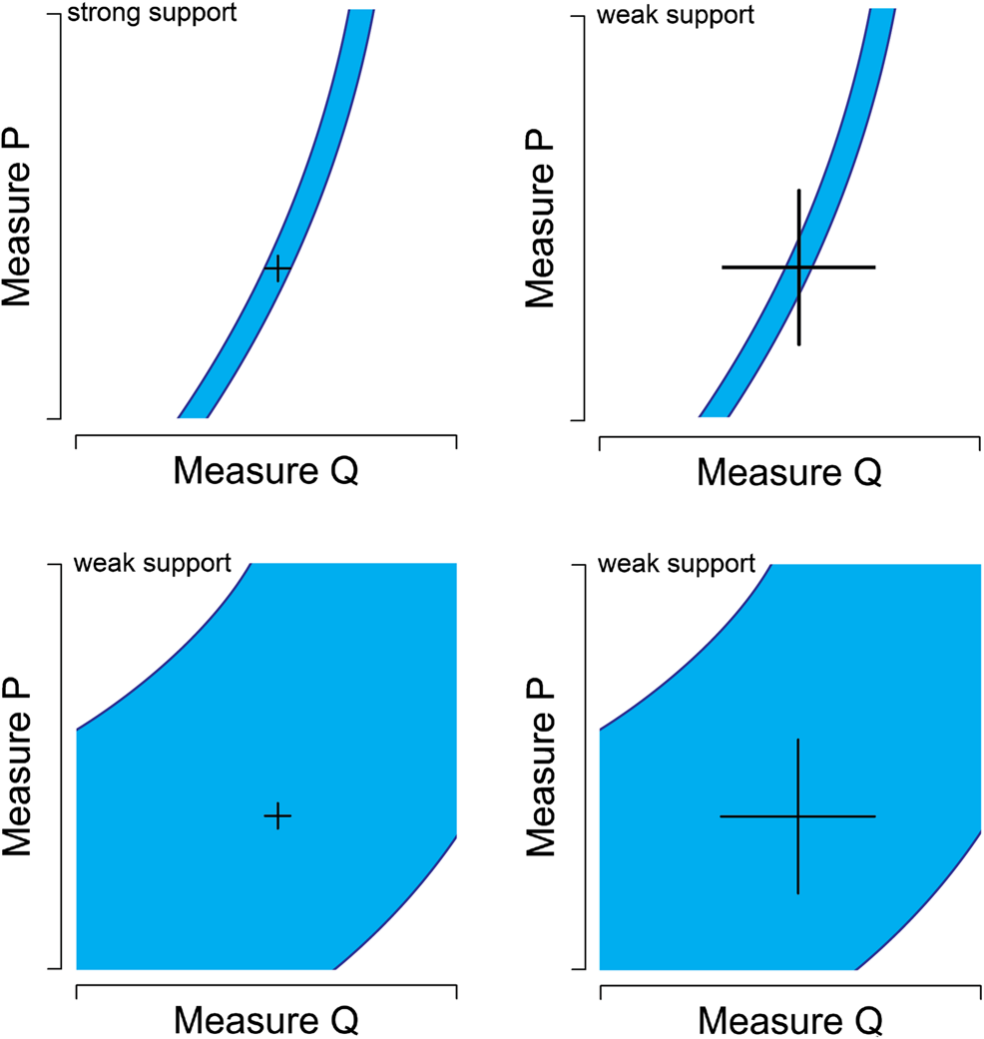
Four possible relations between data and theory. Measures P and Q are both measures of some observable. The axes cover the range of possible values. The highlighted areas indicate the outcomes that are consistent with the theory. Standard errors of the observation are indicated by the error bars. In this example, the theory fits the data, though only when both theory and data are sufficiently constraint (upper left) does this provide significant evidence for the theory (this figure is published under CC-BY 4.0 and is adapted from: Roberts & Pashler, 2000, p. 360)

Figure 3 shows for a theory concerned with the relation between the values of two measures that the data provide strong support only if (a) a narrow range of possible values is consistent with the theory; (b) the data fall in this narrow range; (c) the experimental measurements were precise. These three criteria can be summarized as specificity of predictions, fit and measurement precision.

Vanpaemel (2010, 2020) points out that the Bayesian can express criterion (a) by means of the prior predictive distribution ${\int \limits } p(x|\theta , H) p(\theta |H) d\theta $: all outcomes that fall outside the “core predictions” of a hypothesis *H* (e.g., the data points with the 95%, 99% or 99.99% prior probability mass) are judged to be inconsistent with the theory.^6^ In particular, Vanpaemel (2020) demands that the tested hypothesis do not only rule out possible, but also *plausible* outcomes. This means that possible outcomes that fall outside the X% highest density region as predicted by the hypothesis have some non-negligible probability based on, for instance, prior results (for a more detailed explanation, see ; Vanpaemel, 2020). Only in this case can a hypothesis be tested severely.^7^

Two comments or amendments on Vanpaemel’s account are in order. First, Vanpaemel defines the “plausible” in the requirement of excluding plausible outcomes as independent of the models that are entertained. However, he does not explain how this understanding squares with, or should be reconciled with, the definition of “plausible” via the data prior in the model that encompasses the tested hypotheses. Neither does Vanpaemel offer a clear specification of *how many* plausible outcomes, relative to the overall data space, need to be excluded.

Second, Vanpaemel argues that the threshold for counting outcomes as inconsistent with a model depends on the context: in his running example of the 2020 paper, he chooses 99.99%, but he adds that in other contexts, other thresholds may be possible. Also here, we agree, but we would like to add that we (still) cannot test a hypothesis *H* in isolation, in the sense of “if the actual data fall outside the core predictions, the theory is *H* refuted”. This would amount to relying on Fisher’s infamous disjunction (Fisher, 1956; Cohen, 1994)—either a very improbable event has occurred, or the tested hypothesis must be wrong—and replicating the inverse probability fallacy: since *p*(*E*|*H*) is low, and since we want to explain events in a systematic way, we infer that *p*(*H*|*E*) is low. *A theory must always be tested against a competitor.* Indeed, this aspect of severe testing is underappreciated in Vanpaemel’s account, just as it is missing in Pashler and Roberts’s and Mayo’s account.

From a Bayesian perspective, specificity contributes to severity by means of increasing the diagnosticity or *expected evidential value* of a hypothesis test. This concept can be operationalized as the expected absolute log-Bayes factor^8^ of the experiment (Good, 1950; 1975; 1983; 1979; Lindley, 1956; Nelson, 2005; Schönbrodt and Wagenmakers, 2018; Stefan et al., 2019):

1 $$ \mathbb{E}_{y}\left[|\log \text{BF}_{10}|\right] = \int p(y) |\log \text{BF}_{10}(y)| \text{d}y, $$

where *p* is the prior probability density function over the sample space that encompasses both *H*_0_ and *H*_1_.^9^ The expected absolute log-Bayes factor quantifies the amount of evidence one can expect, for and against combined, where values close to $\mathbb {E}_{y}|\text {log BF}_{10}| = 0$ constitute uninformative tests (i.e., the hypotheses make similar predictions).^10^

When a hypothesis is highly specific, its predictions will in general differ notably from the alternative, and this means that we can expect strong evidential support for either one or the other hypothesis. Furthermore, the expected evidential value is higher for the more restricted hypothesis: it is easier to find evidence against it when it is false, and one obtains more easily evidence for it when it is true. Testing simple, restrictive hypotheses is thus valuable from a Bayesian perspective. Typically—but not always—the most simple, and most severely testable hypothesis is a point null hypothesis *H*_0_ which fixes the parameter of interest to a single number. More generally, there is a systematic connection between the degree to which a hypothesis restricts its parameters, and the degree to which it restricts the range of data consistent with it.^11^ We will now illustrate this reasoning with a simple binomial model.

### An example of specific hypothesis testing

Let us clarify this specification of the Bayes factor with a simple fictitious example. For this example, we have adapted the *informative hypothesis testing* approach (Klugkist & Hoijtink, 2005; Klugkist et al., 2005; Hoijtink, 2011). The Bayes factors reported in this section are calculated with respect to an encompassing model with a uniform prior. We have added the mathematical explication as an Appendix.

In this example, we imagine that military veterans receive either one of two treatments to address post traumatic stress disorder (PTSD). Treatment A is regular psychotherapy (e.g, Cognitive Processing Therapy; Monson et al., 2006). Treatment B is the same psychotherapy but enhanced with regulated dosages of the drug 3,4-Methylene-dioxymethamphetamine (Sessa, 2017).

Based on the theory and previous research, success rates of these treatments should fall in a particular range. The more uncertainties there are in the theory about what could influence the success rate (e.g., inter-patient psychological variability) and the more measurement error one can expect (e.g., low reliability of PTSD assessment), the wider the interval around these expected values that are still considered consistent with the theory.

Specifically, assume that either treatment has a true success rate, *𝜃*_*A*_ and *𝜃*_*B*_. Under an encompassing model these rates can have any value between 0 and 1, *H*_*e*_ : *𝜃*_*A*_ = [0.0,1.0],*𝜃*_*B*_ = [0.0,1.0]. For simplicity, we consider these values equally likely and adopt a uniform prior over *𝜃*_*A*_ and *𝜃*_*B*_ in the encompassing model. The scenario can be modelled as two independent binomial distributions. We start treating participants, randomly divided in equal number to each treatment, and count the number of successful treatments. In this case, for a treatment we would have a specific number of participants *N*_*i*_, where *i* ∈{*A*,*B*}, and the number of successes for this treatment *S*_*i*_ would be determined by the actual value of *𝜃*_*i*_ and sampling error: $S_{i} \sim Bin(N_{i}, \theta _{i})$.

**Fig. 4:**
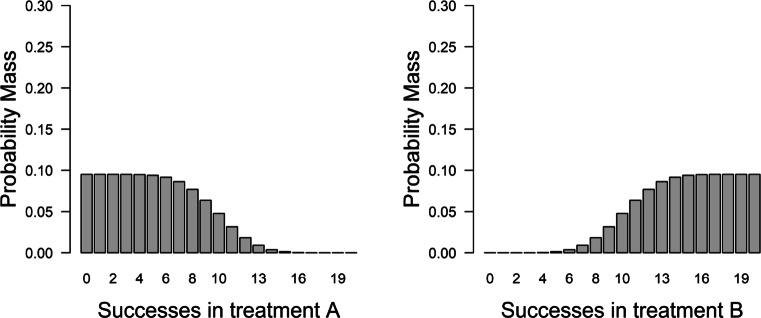
Data predictions for the vague hypothesis. If twenty patients were to be tested in both Treatment A and Treatments B, then according to *H*_*v*_ we would expect to see these numbers of success with these probabilities

We use four scenarios to allow comparison with the four cases in Fig. 3 identified by Roberts and Pashler (2000). Specifically, we consider two hypotheses, one vague and one specific, and two different datasets, one with few and one with many patients. In the first and second scenario, corresponding to the bottom panels in Fig. 3, the hypothesis makes vague predictions: *H*_*v*_ : *𝜃*_*A*_ = [0.00,0.50],*𝜃*_*B*_ = [0.50,1.00].^12^ Thus, *H*_*v*_ takes up 25% of the encompassing model and it predicts that the success rates for Treatment A and B will very likely be below and above 0.5, respectively (see Fig. 4). From Vanpaemel’s perspective, a severe test of this (vague) hypothesis is possible, because plausible outcomes fall outside the highest density region. Specifically, between 14 and 20 successes for treatment A and between 0 and 6 successes for treatment B fall outside the 99% highest density region.

**Fig. 5:**
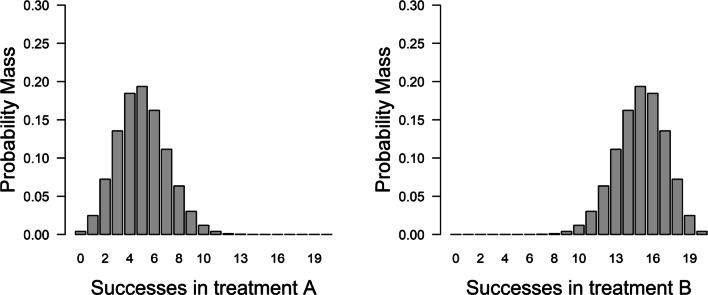
Data predictions for the specific hypothesis. If twenty patients were to be tested in both Treatment A and Treatments B, then according to *H*_*s*_ we would expect to see these numbers of success with these probabilities

In the third and fourth scenario, corresponding to the two top panels in Fig. 3, the hypothesis makes specific predictions and strongly restricts the possible parameter values: *H*_*s*_ : *𝜃*_*A*_ = [0.20,0.30],*𝜃*_*B*_ = [0.70,0.80]. Thus, *H*_*s*_ takes up 1% of the encompassing model and it predicts that success rates between 0.2 and 0.3 for Treatment A and between 0.7 and 0.8 for Treatment B are most probable while success rates above and below those values are increasingly unlikely (see Fig. 5). From Vanpaemel’s perspective, a severe test of this (specific) hypothesis is possible, because plausible outcomes fall outside the highest density region. Specifically, 0 and between 12 and 20 successes for treatment A and between 0 and 9 and 20 successes for treatment B fall outside the 99% highest density region.^13^

**Fig. 6:**
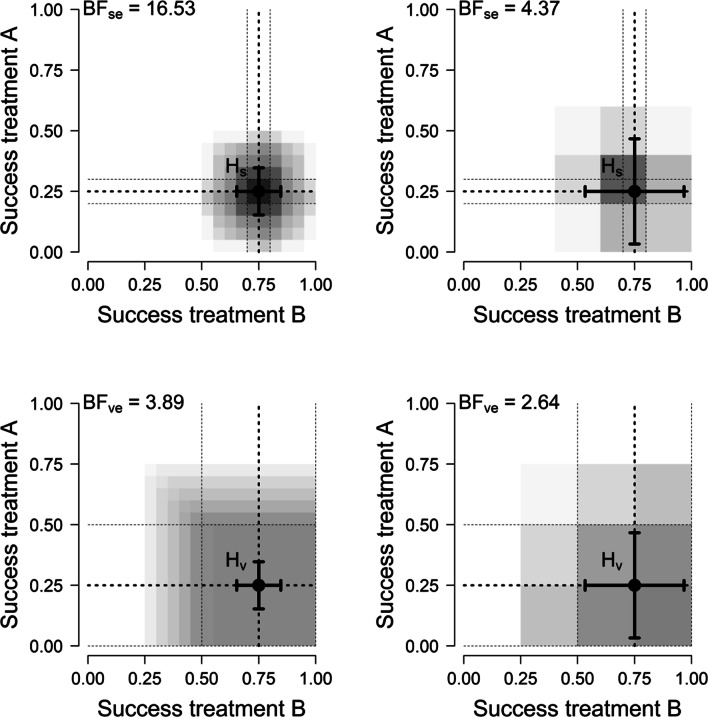
Four relations between hypothesis and data. The top two graphs show the results of specific hypothesis *H*_*s*_. The bottom two graphs show the results of the vague hypothesis *H*_*v*_. The gradient gray areas depict the probabilities mass with respect to the hypotheses’ predicted outcomes (a top view of Figs. 5 and 4). The dotted-lines in gray visualize the hypotheses’ restrictions on the parameter values. The point estimates and standard errors are visualized as black crosses. The graphs in the left column display the results of the large data set (*N*_*i*_ = 20) and the graphs in the right column display the results of the small data set (*N*_*i*_ = 4). In this example, the data align perfectly with the hypotheses. The evidential support for the hypotheses in comparison to the encompassing model is quantified as Bayes factors (top-left of each plot). From top-left to bottom-right, these Bayes factors are 16.53, 4.37, 3.89, and 2.64 respectively

These hypotheses are compared on two data sets. A small data set of four participants per treatment, *N*_*A*_ = *N*_*B*_ = 4 represents the two right panels of the Roberts and Pashler (2000) quartet. A relatively large data set of twenty participants per treatment, *N*_*A*_ = *N*_*B*_ = 20, represents the two left panels of the Roberts and Pashler (2000) quartet. If the data align well with each hypothesis (e.g., $S_{A}=\frac {1}{4} N_{A}$ and $S_{B}=\frac {3}{4} N_{B}$), the specific hypothesis *H*_*s*_ is better supported than the vague hypothesis *H*_*v*_. It is even the case that the specific hypothesis is better supported by the small data set (BF_*se*_ = 4.37) than the vague hypothesis is supported by the large data set (BF_*ve*_ = 3.89). As shown in Fig. 6, this scenario qualitatively reproduces the figure of Roberts and Pashler (2000).

**Fig. 7:**
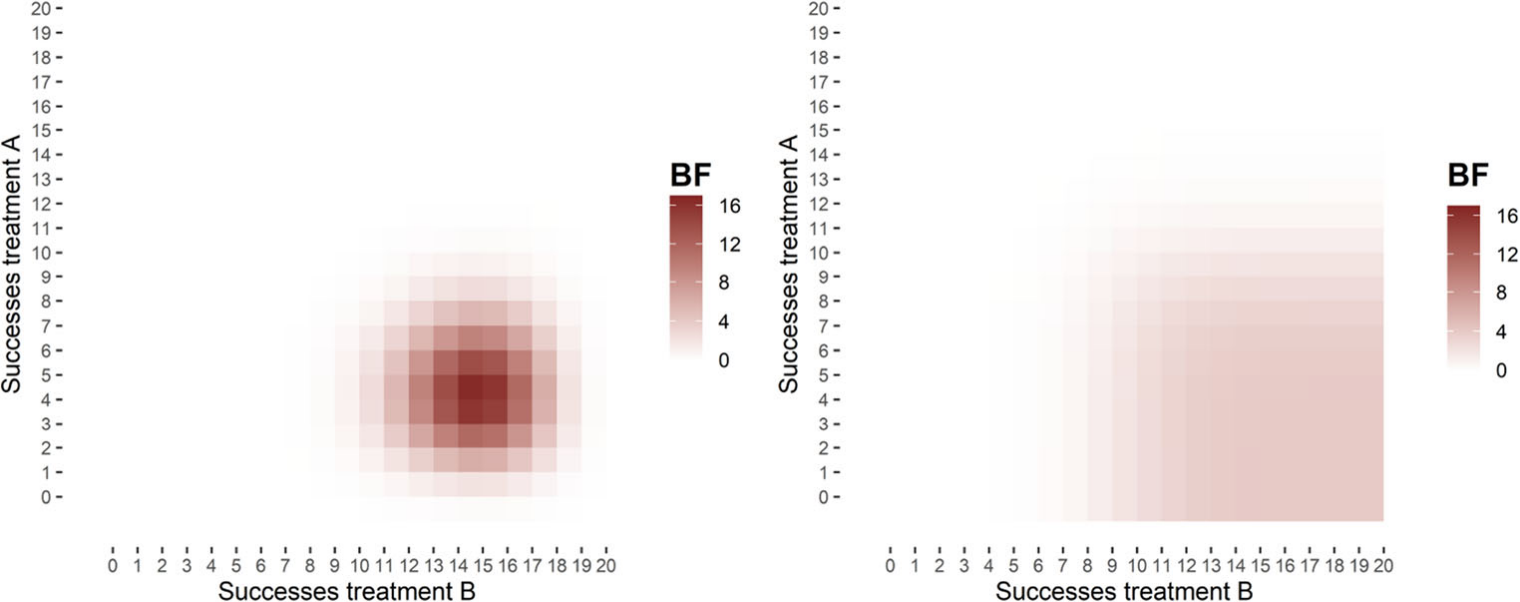
Specific hypotheses can yield more evidence than vague hypotheses, though in most situations this is evidence against the hypothesis. The graphs displays the size of the Bayes factor with respect to possible combinations of outcomes for the specific hypothesis *H*_*s*_ (left) and the vague hypothesis *H*_*v*_ (right). The *x*-axis and *y*-axis indicate, for treatment A and treatment B respectively, the number of successful treatments (*S*_*i*_) out of the 20 participants that are treated (*N*_*i*_ = 20). The values within the lattice indicate the evidence for the hypothesis that results from the particular combination of *S*_*A*_ and *S*_*B*_. This evidence is quantified in terms of the Bayes factor for the hypothesis (left: specific, right: vague) with respect to the encompassing model. The size of the Bayes factor is also indicated as the intensity in color

However, the benefit of a specific hypothesis when right comes at a cost when wrong. As is visible in Fig. 7, *H*_*s*_ only has a small amount wiggle room and the Bayes factor quickly drops towards zero when the number of successes deviates from the predicted range, while *H*_*v*_ has a large area to move in with respect to possible success rates that support it.

This is also reflected in the expected evidential value of the experiment in the four situations of the example. As explained in the previous section (see Eq. 1), the expected absolute log-Bayes factor describes the evidential value one can expect either for or against the specific hypothesis in relation to the encompassing model, where the expectation is taken over all possible data *y*:

$$ \mathbb{E}\left[|\log \text{BF}_{se}|\right] = \int p(y) |\log \text{BF}_{se}(y)| \text{d} y $$

where *p*(*y*) is

$$ p(y) = p({H}_{s}) p(y \mid {H}_{s}) + p({H}_{e}) p(y \mid {H}_{e}) $$

and *p*(*y*|*H*) is

$$ p(y \mid {H}) = \int p(y \mid \theta, {H}) g(\theta \mid {H})  \text{d} \theta $$

In this example, the computation of $\mathbb {E}\left [|\log \text {BF}_{se}|\right ]$ comes down to taking the average of absolute log Bayes factors over all possible treatment outcomes for both Treatment A and Treatment B, weighted by the marginal probability of these treatment outcomes according to *H*_*s*_ and the encompassing model. Analogously for $\mathbb {E}\left [|\log \text {BF}_{ve}|\right ]$.

As can be gleaned from Fig. 7, there is substantial similarity between the vague hypothesis and the encompassing model, in the sense that a quite large set of possible outcomes is explained to a similar degree by either hypothesis. This property decreases the expected evidential value of the experiment. When we move to specific hypothesis, on the other hand, there are striking differences between the outcomes expected under the hypothesis and the encompassing model. This is also reflected in the expected evidential values: $\mathbb {E}_{y}\left [|\log \text {BF}_{se}|\right ] > \mathbb {E}_{y}\left [|\log \text {BF}_{ve}|\right ]$. The expected evidential value is highest for the specific hypothesis with the predetermined sample size of 20 per treatment, $\mathbb {E}[|\log \text {BF}_{se}|]=6.32$. This is followed by the vague hypothesis with *N*_*i*_ = 20 per treatment, $\mathbb {E}[|\log \text {BF}_{ve}|]=3.14$; the specific hypothesis with *N*_*i*_ = 4 per treatment, $\mathbb {E}[|\log \text {BF}_{se}|]=1.61$; and the vague hypothesis with *N*_*i*_ = 4 per treatment, $\mathbb {E}[|\log \text {BF}_{ve}|]=1.25$.^14^

Summing up, the evidence is much more telling and discriminatory when testing specific hypotheses (cf. Etz et al., 2018). From a Bayesian perspective, these numbers show why severe testing is important for inference and decision-making, and what specific hypotheses with contrastive predictions contribute to the value of an experiment. We have examined the case of testing a nested hypothesis against an encompassing model, but the case generalizes straightforwardly to other statistical models, and specifically, to models with contrastive, mutually exclusive hypotheses.

### Error control and contrastive hypotheses in Bayesian inference

For the Bayesian, it is essential that theory testing, and scientific method as a whole, are *comparative*. Experiments can be probative and severe only when contrasting a hypothesis *H*_0_ to an explicit alternative *H*_1_. And this alternative must be specified in the light of our best scientific knowledge, i.e., in the light of our prior expectations. Specifically, the Bayesian answers the question “... what constitutes a good theory?” (Lindley, 2006, p. 196) in terms of its potential to yield high likelihood ratios in testing the theory against its negation:

[...] A good theory is one that makes lots of predictions that can be tested, preferably predictions that are less probable were the theory not true. [...] what is wanted are data that are highly likely when the theory is true, and unlikely when false. A good theory cries out with good testing possibilities (Lindley, 2006, p. 197, notation changed).

That experiments should be as contrastive as possible has already been highlighted in the previous section. It is especially important because the more two hypotheses make contrasting predictions, the better can we limit the *probability of misleading evidence*, along the lines of the Lindley) (see also Royall 2000, for a semi-Bayesian, likelihoodist treatment), and assess whether the test was severe.

One Bayesian tool for making this assessment is a Bayes Factor Design Analysis (BFDA; Schönbrodt & Wagenmakers 2018, see also https://shinyapps.org/apps/BFDA/). Before collecting data, the experimenter decides on a threshold for when a Bayes factor counts as evidence for a hypothesis, e.g. BF_10_ > 3 or BF_10_ < 1/3. Of course, one can set more stringent requirements as one desired (e.g., BF_10_ = 10, the threshold for strong evidence). Suppose the alternative hypothesis *H*_1_ is in fact true. The BFDA then calculates

- the probability of obtaining positive evidence for *H*_1_ (e.g., BF_10_ > 3);
- the probability of obtaining inconclusive evidence (e.g., 1/3 ≤BF_10_ ≤ 3); and
- the probability of obtaining misleading evidence for *H*_0_ (e.g., BF_10_ < 1/3).

**Fig. 8:**
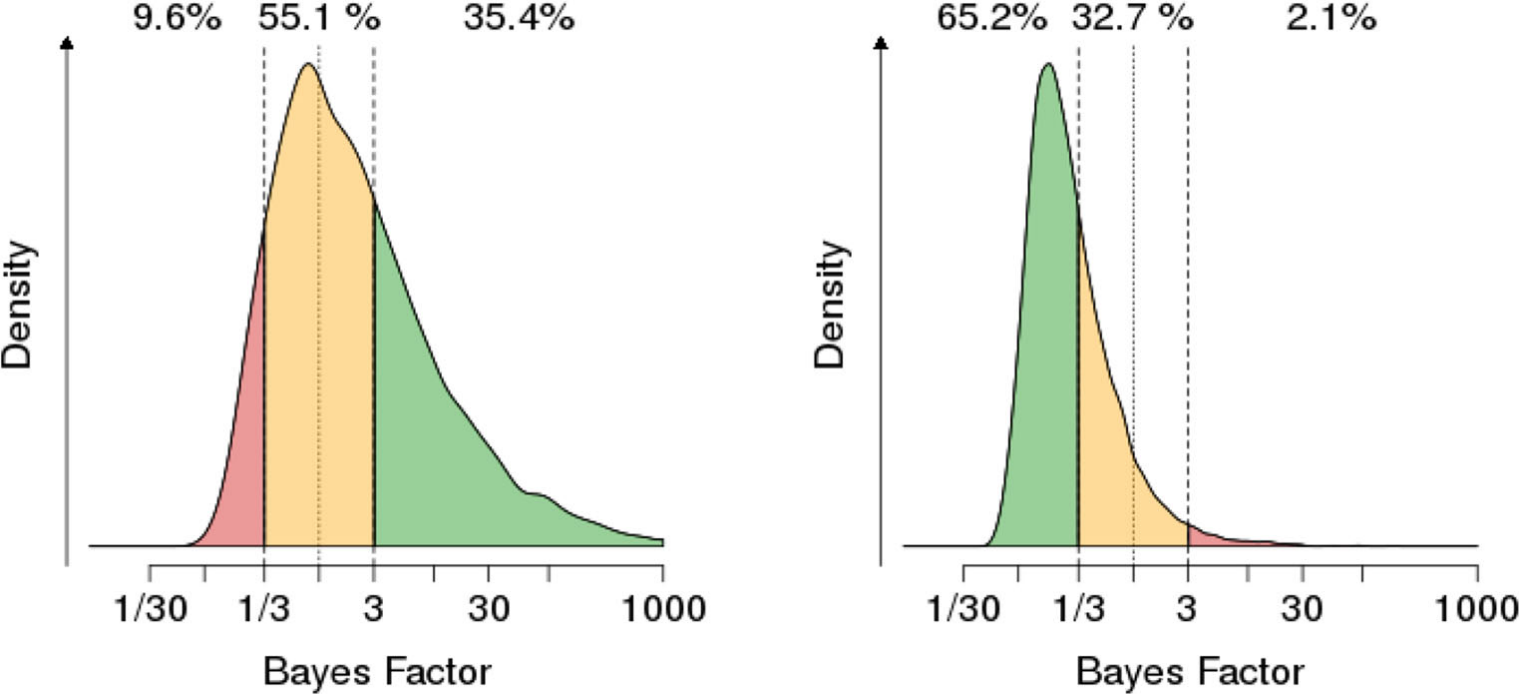
Graphical illustration of the distribution of Bayes factors and probability of misleading evidence in the Bayesian framework for N = 36, a two-sided *t*-test and hypothesized effects of *d* = 0 and *d* = 0.4. Figure produced with the BFDA app https://shinyapps.org/apps/BFDA/ based on Schönbrodt and Wagenmakers (2018)

Identically, one can define the probability of obtaining positive evidence for *H*_0_, and misleading evidence for *H*_1_ (when *H*_0_ is true). A graphical illustration is given in Fig. 8 for a two-sided *t*-test with a moderate group size *N* = 36 in both groups. The probability of finding misleading evidence for *H*_0_ when *H*_1_ is true is relatively large: 9.6%. Moreover, the most likely result is actually to obtain inconclusive evidence (55.1%). Certainly such an experiment controls error rates (here defined not as Type I or Type II errors, but in the above sense of finding misleading evidence) poorly and cannot be counted as a severe test of the competing hypotheses.

**Fig. 9:**
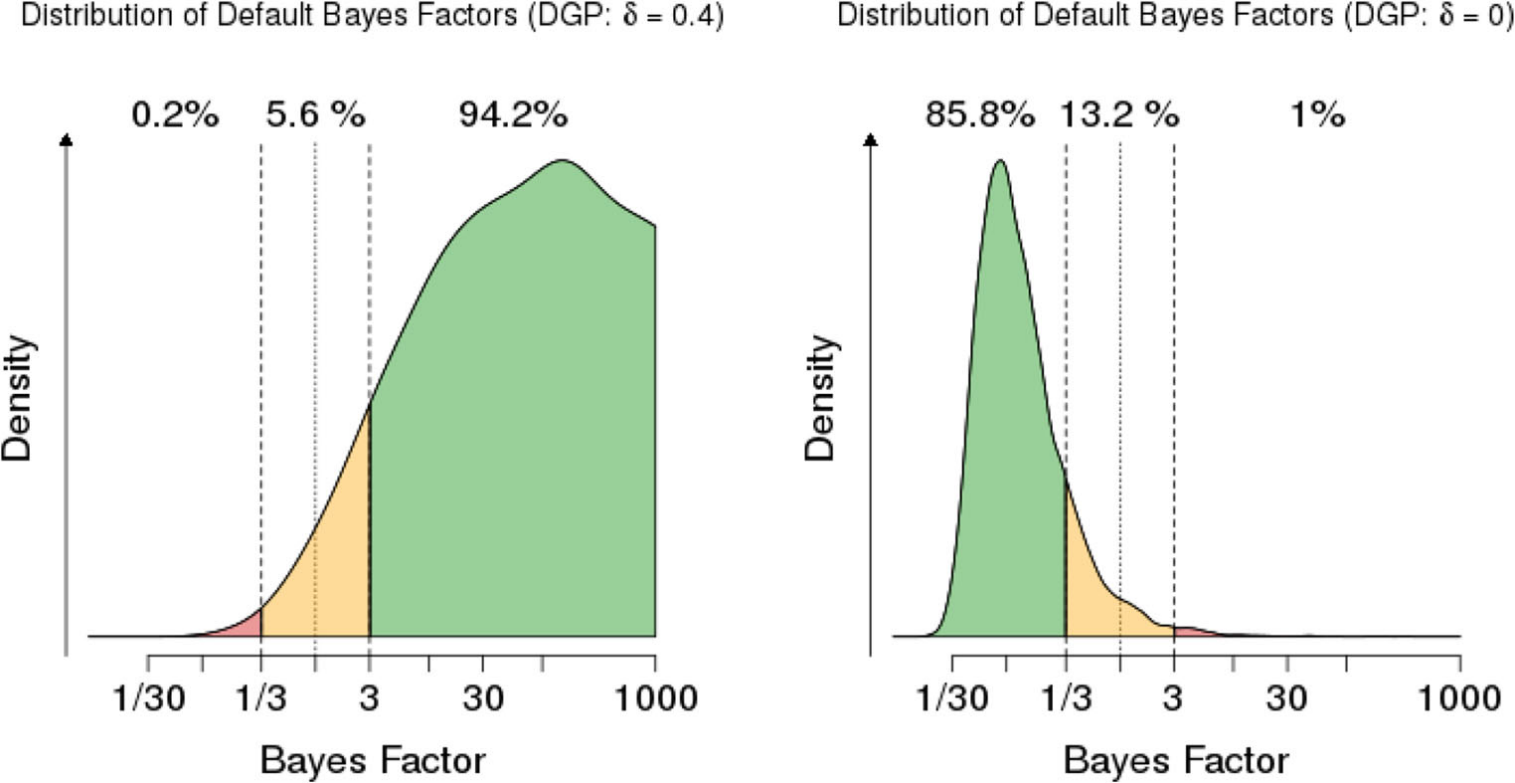
Graphical illustration of the distribution of Bayes factors and probability of misleading evidence in the Bayesian framework for N = 190, a two-sided *t*-test and hypothesized effects of *d* = 0 and *d* = 0.4. Figure produced with the BFDA app https://shinyapps.org/apps/BFDA/ based on Schönbrodt and Wagenmakers (2018)

When the sample size increases, the impact of sampling variability is reduced and this facilitates the control of the probability of misleading evidence. With *N* = 190, for example, these rates drop to 0,2% and 1%, respectively. See Fig. 9.

This shows that error rates are no unique feature of frequentist statistics: the Bayesian has analogous tools to calculate the probability of misleading evidence and to construct his (or her) experiment in a way to control for the probability of misleading evidence. This is exactly what Mayo alludes to in her Severity Principle, which we quote again for convenience:

**Severity Principle (strong):** We have evidence for a claim *C* just to the extent it survives a stringent scrutiny. If *C* passes a test that was highly capable of findings flaws or discrepancies from C, and yet none or few are found, the passing result, x, is evidence for C.

Reinterpreting Mayo from a Bayesian viewpoint, we have evidence for the claim *C* if and only if (a) we observe a Bayes factor in favor of *C* beyond a context-sensitive threshold, and (b) the probability of finding misleading evidence for *C* (under the assumption that *C*’s competitor is true) is low. The Shiny apps and the R package for a Bayes Factor Design Analysis make the implementation of this planning explicit and easy to handle for an experimenter. There is thus no reason why a Bayesian needs to give up an appreciation of severity in terms of error control. What is more, in the light of the challenges inherent to Mayo’s own operationalization of her Severity Principle, one could even draw the conclusion that Bayesian inference is suited best to implement her philosophical stance into experimental practice.

## Discussion

This article presents a Bayesian proposal on how to accommodate the concept of severity when testing statistical hypotheses. It provides a translation of Popper’s (1959/2002) falsificationist philosophy and the intuitive impressiveness of risky predictions in terms of a test of specific hypotheses. The specificity of a hypothesis is defined by the degree to which predictions are spread out across the sample space, while evidence is defined by relative predictive performance, that is, how much probability mass is allocated to the data under the competing hypotheses (i.e., the Bayes factor).

A complex or vague hypothesis spreads out its predictive mass across a wide range of options, and by hedging its bets will lose out against a more restrictive hypothesis that makes a more precise (and accurate) prediction. The more precise the predictions from the competing hypotheses, the higher the expected diagnosticity, and the more severe the test. This approach clearly quantifies Popper’s idea of evaluating theories on the basis of their empirical content and degree of falsifiablity (Popper, 1959/2002). In the specific case of a parameter space whose regions correspond to different statistical hypotheses, the specificity of a hypothesis can often be measured by the proportion of the parameter space it occupies (weighted with the prior probability density; cf. Appendix).

Popper and Bayes can thus be reconciled: the evaluation of hypotheses in terms of Bayes factors is influenced by their specificity and Bayesian inference has the conceptual resources to reward specific predictions. Notably, obtaining this conclusion does *not* require any blending of Bayesian and frequentist inference; our account stays faithful to the principles of subjective Bayesianism. While error statistics postulates severe testing as a key virtue of statistical inference, our approach also *explains* why severity matters for conducting efficient hypothesis tests and making good decisions.

### Advantages of specific hypothesis testing

There are several clear advantages to testing specific hypotheses in a Bayesian framework. First and foremost, it is to the researchers’ benefit to make more specific predictions. As outlined earlier, the more specific the hypothesis is, the more evidence one can expect: either more evidence is obtained from the same amount of data, or less data are needed for the same amount of evidence. This is in stark contrast with orthodox frequentist methods where “more evidence” requires more data if the discrepancy between null hypothesis and alternative hypothesis is fixed. In experimental psychology, demonstrations of the benefit of informed/specific Bayes factor hypothesis tests includes Vohs et al., (in press), Gronau et al., (2017), Ly et al., (2019) and in particular the work of Zoltan Dienes (e.g., Dienes, 2008; 2011; 2014; 2016; 2019). For cognitive models, the value of informed prior distributions has been highlighted by Vanpaemel and Lee (2012) and Lee and Vanpaemel (2018).^15^ All in all, unlike frequentists, Bayesian can directly integrate theoretical expectations into the premises of a statistical inference and use them to test hypotheses severely.

Second, our account makes explicit why vague predictions lack diagnostic value. This is most clearly expressed in cases where (almost) everything is consistent with the theory. In such cases where all parameter values are allowed by the theory, one can never expect to obtain strong evidence either in favor or against the theory. Only when the theory is restrictive, strong confirming or undermining evidence can plausibly be expected. Consequently, evidence is limited by the specificity of the hypothesis. Intuitively, the possible evidence in favor of a theory should be limited by its strength and how well it is tested. The specific hypothesis testing approach provides this feature, because the maximum amount of evidence in terms of the Bayes factor is one divided by the specificity of the hypothesis.

Third, our approach extends the treatment of severity by Vanpaemel (2019; 2020), who argues that predictions are risky, and tests severe, only if the tested hypothesis rules out plausible outcomes a priori. Vanpaemel’s approach is so far qualitative, and the exclusion of plausible outcomes can be taken as a minimal requirement of a severe test as we did in the example discussed in the previous section.

Fourth, our approach to severity can be extended to the research planning phase. Specifically, the specificity of one’s hypothesis, or the restrictiveness of one’s theory, can inform the researcher about the sample size required to be fairly certain of strongly supporting or contradicting results. Vice versa, in the case where the sample size is predetermined, the researcher can infer how specific the tested hypothesis needs to be in order to be fairly certain of obtaining high degrees of confirmation or disconfirmation. Explicitly, our approach could be integrated into the Bayes Factor Design Analysis discussed in the previous section (BFDA; Schönbrodt & Wagenmakers, 2018; Stefan et al., 2019). Given background assumptions concerning the rival models and the data-generating process, BFDA provides either the distribution of Bayes factors for a fixed sample size, or the distribution of sample sizes to obtain a fixed Bayes factor. The use of more specific models will affect the BFDA such that the same data will become more diagnostic: a fixed sample size is projected to yield more evidence, and a given level of evidence is reached with smaller samples.

Similarly, in Adaptive Design Optimization (ADO; e.g., Ahn et al., 2020; Myung et al., 2013) the next stimulus to be presented is determined by maximizing expected information gain. As the observations accumulate, the rival hypotheses become increasingly specific (i.e., their constituent posterior distributions become more peaked), and therefore easier to discriminate. Our research thus connects to the growing literature that considers Bayesian inference not only a method of hypothesis evaluation, but also uses it in planning and designing experiments that are both reliable and efficient.

### Disclaimers and conclusion

Our approach is susceptible to any objection that may be raised against Bayesian statistics as a whole (e.g., Moyé, 2008; Senn, 2011; Mayo, 2010; 2018). Our claim here is just that the epistemic value of severity is not a compelling argument for preferring frequentist to Bayesian statistics. Severity is a notoriously elusive and hard-to-quantify concept in the frequentist paradigm, too, and the state-of-the-art explications are arguably unsatisfactory. At the same time, it is possible to give a Bayesian account of the evidential value of severity. We hope that our approach provides a useful tool that can fill an empty spot in the researcher’s toolbox of statistical methods, specifically with regard to the role of severe testing Bayesian inference. At the very least, we expect that this paper will inspire criticism, which might stimulate fruitful debate on how theories can show their probative value irrespective of the preferred statistical paradigm.

## Data Availability

The *R* code for calculating the Bayes factors is available at https://osf.io/3cdyx/.

## Appendix: specific hypothesis testing with encompassing priors

The Bayesian *informative hypothesis testing* approach (e.g., Klugkist et al., 2005; Klugkist & Hoijtink, 2005) can be used to illustrate how Bayesian inference incorporates the concept of severity as the ability of an experimental design to discriminate the hypotheses at hand. Within this approach, each hypothesis to be tested is derived from a more general *encompassing* model. This model consists of all pertinent parameters free from any constraints (e.g., *H*_*e*_ : *μ*_*a*_,*μ*_*b*_); all specific hypotheses are nested in this model (e.g., *H*_0_ : *μ*_*a*_ = *μ*_*b*_, *H*_1_ : *μ*_*a*_ > *μ*_*b*_, *H*_2_ : *μ*_*a*_ < *μ*_*b*_). Only a prior for the parameters in the encompassing model needs to be specified and the priors from the nested hypotheses follow from this encompassing prior. Specifically, the hypotheses occupy particular sections of the parameter space, allotting each hypothesis a segment of the encompassing prior (for further explanation of the encompassing prior, see ; Klugkist & Hoijtink, 2005).

We can denote the encompassing model as *H*_*e*_ and the encompassing prior as *g*(***𝜃***|*H*_*e*_), where ***𝜃*** is the vector of parameters of interest. The prior distribution of any hypothesis *H*_*i*_ nested in *H*_*e*_ can be obtained from the encompassing prior by restricting the parameter space according to the limits of the hypothesis, given by

$$ g(\boldsymbol{\theta} \mid {H}_{i}) = \frac{g(\boldsymbol{\theta} \mid {H}_{e})I_{{H}_{i}}(\boldsymbol{\theta})}{\int g(\boldsymbol{\theta} \mid {H}_{e}) I_{{H}_{i}}(\boldsymbol{\theta})d\boldsymbol{\theta}}. $$

Here, $I_{{H}_{i}}(\boldsymbol {\theta })$ is an indicator function which equals 1 if the parameter value is within the limits of *H*_*i*_ and 0 if it is outside the limits of *H*_*i*_.

The evaluation of the hypothesis is based on the Bayes factor. Specifically, support provided by the data for one versus another hypothesis is quantified as the ratio of marginal likelihoods of hypotheses (Kass & Raftery, 1995). For data *x* and hypotheses *H*_*i*_ and *H*_*e*_, the Bayes factor (BF) is

$$ \text{BF}_{ie} = \frac{p(x \mid {H}_{i})}{p(x \mid {H}_{e})}, $$

that is, the quotient of the marginal likelihoods for the hypotheses *H*_*i*_ and *H*_*e*_. Due to Bayes’ Theorem

$$ p(\boldsymbol{\theta} \mid x, {H}) = \frac{ p(x \mid \boldsymbol{\theta}, {H})g(\boldsymbol{\theta} \mid {H})}{p(x\mid {H})}, $$

the marginal likelihood can, for any value of the unknown parameter ***𝜃*** consistent with *H*, also be written as

$$ p(x\mid {H}) = \frac{ p(x \mid \boldsymbol{\theta}, {H})g(\boldsymbol{\theta} \mid {H})}{ p(\boldsymbol{\theta} \mid x, {H})}. $$

In this formulation, the denominator is the posterior density of ***𝜃*** under model *H*_*i*_ and the numerator is the product of the likelihood function and the prior distribution. As a consequence, the Bayes factor of *H*_*i*_ and *H*_*e*_ can be written as

2 $$  \text{BF}_{ie} = \frac{ p(x \mid {H}_{i})}{p(x\mid{H}_{e})} = \frac{ p(x\mid\boldsymbol{\theta}, {H}_{i}) g(\boldsymbol{\theta} \mid {H}_{i}) / p(\boldsymbol{\theta} \mid x, {H}_{i})}{ p(x \mid \boldsymbol{\theta}, {H}_{e}) g(\boldsymbol{\theta} \mid {H}_{e}) / p(\boldsymbol{\theta} \mid x, {H}_{e})}. $$

If we then suppose, more specifically, that the value ***𝜃*** is allowed by *H*_*i*_, (2) delivers

$$ \text{BF}_{ie} = \frac{ g(\boldsymbol{\theta} \mid {H}_{i})p(\boldsymbol{\theta} \mid x, {H}_{e})}{ g(\boldsymbol{\theta} \mid {H}_{e}) p(\boldsymbol{\theta} \mid x, {H}_{i})}. $$

Through *H*_*i*_ being nested in *H*_*e*_, the densities *g*(***𝜃***|*H*_*i*_) and *p*(***𝜃***|*x*,*H*_*i*_) can be rewritten as follows:

$$ \begin{array}{@{}rcl@{}} g(\boldsymbol{\theta}  |  {H}_{i}) &= c_{i} \times g(\boldsymbol{\theta}  |  {H}_{e}) \\ p(\boldsymbol{\theta}  |  x, {H}_{i}) &= f_{i} \times p(\boldsymbol{\theta}  |  x, {H}_{e}), \end{array} $$

where *c*_*i*_ and *f*_*i*_ are constants. Thus, *H*_*i*_’s prior and posterior densities can be given in terms of the densities of the encompassing model *H*_*e*_. 1/*c*_*i*_ denotes how much of the prior distribution of *H*_*i*_ is in agreement with the encompassing model *H*_*e*_, and 1/*f*_*i*_ denotes the same quantity for the posterior distribution. 1/*c*_*i*_ is therefore a natural measure of the complexity of the nested model *H*_*i*_, 1/*f*_*i*_ of its post hoc fit. Since *H*_*i*_ is nested within *H*_*e*_, we can write the Bayes factor simply as

$$ \text{BF}_{ie} = \frac{1/f_{i}}{1/c_{i}} = \frac{\text{fit}}{\text{complexity}} $$

From this specification of the Bayes factor, it follows that good model fit is not enough as evidence for one’s hypothesis. Near perfect fit is easily reached when close to all possible parameter values and functional forms are allowed. Only when one’s hypothesis is restrictive enough that it occupies only a small fraction of possible parameter values and functional forms a priori, though still is in accordance with the data a posteriori, does one have evidence in favor of the hypothesis under consideration when supporting observations are made.

## Footnotes

1 For an argument that unverifiable hypotheses are essential for scientific progress see e.g., Poincaré (1913).

2 We recognize that in principle it is easier to scrutinize a precise prediction than it is to scrutinize a vague prediction. However, precision is not a requirement for a stringent test as Mayo (2018) defines it.

3 Actually, *H*_0_ is rejected in favor of the standard alternative hypothesis *H*_*a*_ : *μ* > *μ*_0_, to keep the parameter space neatly partitioned, but this does mean that the data are consistent with *C*.

4 For convenience, the *SEV* function is evaluated at *μ* = *μ*_0_ + *δ*, because the probability value that it produces will be even greater for *μ* < *μ*_0_ + *δ* (for detailed explanation, see Mayo 2018, p. 144).

5 For convenience, Mayo rounds 151.96 up to 152.

6 This idea is similar to using credible intervals based on regions of highest posterior density (ROPE) in order to draw inferences from Bayesian hypothesis tests (Kruschke, 2018; Kruschke & Liddell, 2018).

7 For Vanpaemel, the prior probability distribution for the model parameters is an indispensable element of evaluating the severity of a tests, since the data prior depends on the prior over the parameter space: “Wide data priors make for lenient tests; narrow data priors imply strong tests” (142 ; Vanpaemel, 2020). Since narrow data priors are usually obtained by means of choosing specific hypotheses that occupy only a small range of the parameter space, severe testing is also connected to specificity on his account.

8 Nelson (2005) objects to the use of the expected log-Bayes factor as a measure of diagnostic value on a variety of reasons, of which we recapitulate the two most important ones: (1) it takes infinite value if an outcome is *logically excluded* by *H*_0_; (2) the lack of sensitivity to the prior probability of the hypotheses is not psychologically adequate, as he seeks to demonstrate using a series of cognitive experiments.

9 This expression does not depend on the choice of *H*_0_ vs. *H*_1_ since $|\log BF_{10}| = |\log BF_{10}|$.

10 The idea of taking an expectation over the sample space may look alien to Bayesian inference, but it is a well-established part of the modern Bayesian’s toolbox: see de Finetti (1974, pp. 90-91), Pérez and Berger (2002) and O’Hagan and Forster (2004, pp. 166-167).

11 Popper notes that a hypothesis is the more testable, and the more restrictive on the data, the simpler it is, where simplicity is defined as paucity of parameters (Popper, 1959/2002, pp. 126–128 and 392–394).

12 Locations of these hypotheses are chosen for symmetry; not much changes when different locations are used.

13 A case could be made that 0 and 20 successes are implausible outcomes, because they fall outside the highest density regions for both treatments. However, this is irrelevant for deciding whether or not the hypothesis can be tested severely according to Vanpaemel (2020).

14 The example suggests that point hypotheses are easier to test severely than composite hypotheses, but this holds only *ceteris paribus* and cannot be generalized without qualification.

15 Vohs et al., (in press) assign a positive-only Gaussian prior for effect size with mean .30 and standard deviation .15 – the Vohs prior. Gronau et al., (2017) assign to effect size a positive-only *t*-distribution with location 0.350, scale 0.102, and three degrees of freedom – the Oosterwijk prior, see (Gronau et al., 2020). Finally, Ly et al., (2019) outline a test to assess replication success, where the posterior distribution of the original experiment functions as the prior distribution of the replication experiment; see also Verhagen and Wagenmakers (2014).
